# Cancer-associated mutations in protein kinase C theta are loss-of-function

**DOI:** 10.1042/BCJ20240148

**Published:** 2024-06-10

**Authors:** Stefanie J. Hodapp, Nathan Gravel, Natarajan Kannan, Alexandra C. Newton

**Affiliations:** 1 Department of Pharmacology, University of California, San Diego, La Jolla, CA 92093, U.S.A.; 2 Biomedical Sciences Graduate Program, University of California, San Diego, La Jolla, CA 92093, U.S.A.; 3 Department of Biochemistry and Molecular Biology and Institute of Bioinformatics, University of Georgia, Athens, GA 30602, U.S.A.

**Keywords:** cancer, enzyme activity, protein kinase C theta, signaling

## Abstract

The Ca^2+^-independent, but diacylglycerol-regulated, novel protein kinase C (PKC) theta (θ) is highly expressed in hematopoietic cells where it participates in immune signaling and platelet function. Mounting evidence suggests that PKCθ may be involved in cancer, particularly blood cancers, breast cancer, and gastrointestinal stromal tumors, yet how to target this kinase (as an oncogene or as a tumor suppressor) has not been established. Here, we examine the effect of four cancer-associated mutations, R145H/C in the autoinhibitory pseudosubstrate, E161K in the regulatory C1A domain, and R635W in the regulatory C-terminal tail, on the cellular activity and stability of PKCθ. Live-cell imaging studies using the genetically-encoded fluorescence resonance energy transfer-based reporter for PKC activity, C kinase activity reporter 2 (CKAR2), revealed that the pseudosubstrate and C1A domain mutations impaired autoinhibition to increase basal signaling. This impaired autoinhibition resulted in decreased stability of the protein, consistent with the well-characterized behavior of Ca^2+^-regulated PKC isozymes wherein mutations that impair autoinhibition are paradoxically loss-of-function because the mutant protein is degraded. In marked contrast, the C-terminal tail mutation resulted in enhanced autoinhibition and enhanced stability. Thus, the examined mutations were loss-of-function by different mechanisms: mutations that impaired autoinhibition promoted the degradation of PKC, and those that enhanced autoinhibition stabilized an inactive PKC. Supporting a general loss-of-function of PKCθ in cancer, bioinformatics analysis revealed that protein levels of PKCθ are reduced in diverse cancers, including lung, renal, head and neck, and pancreatic. Our results reveal that PKCθ function is lost in cancer.

## Introduction

The novel protein kinase C (PKC) isozyme, PKC theta (θ), is most abundantly expressed in hematopoietic cells where it transduces signals resulting in T lymphocyte and platelet activation [1–4]. Priming of naïve T cells requires two signals, antigen engagement with the T cell receptor and CD28 co-stimulation [5,6]. This event triggers the translocation of PKCθ to the immunological synapse where it forms a complex with CD28 [7–9]. This interaction is essential for proper CD28 signaling, leading to activation of transcription factors, AP-1, NFAT, and NF-κB, and ultimately T cell activation [10–14]. Although PKCθ signaling has been almost exclusively studied in immune signaling, increasing evidence suggests that PKCθ may be involved in cancer where its expression is consistently reduced compared with healthy tissue [15]. More than 200 unique cancer-associated somatic mutations have been identified in PKCθ across a variety of cancer types, raising the question of their functional relevance. Additionally, analysis of these mutations could provide insight into whether loss or gain of PKCθ function is associated with cancer, in turn informing on whether to target this highly druggable kinase as an oncogene or as a tumor suppressor.

PKC is a family of serine/threonine protein kinases whose members are activated by binding both Ca^2+^ and diacylglycerol (DG) (conventional PKC), by binding DG alone (novel PKC), or by protein:protein interactions (atypical PKC) to regulate diverse processes [16]. All PKC isozymes comprise an N-terminal regulatory region that constrains the activity of a kinase domain, which is further regulated by a C-terminal tail [17]. The subfamily of novel PKC (nPKC) isozymes, which includes PKCθ, contains in their regulatory moiety a Ca^2+^-insensitive C2 domain followed by an autoinhibitory pseudosubstrate segment linked to tandem C1 domains (C1A-C1B). The C2-pseudosubstrate-C1A domains function to autoinhibit the kinase, and the C1B domain is the functional DG sensor [18–20]. Generation of DG engages nPKC isozymes on DG-rich membranes (typically plasma membrane and Golgi), resulting in release of the autoinhibitory pseudosubstrate to effect downstream signaling. Interaction with specific protein scaffolds also serves to localize PKC signaling; in the case of PKCθ, interaction with lymphocyte-specific protein tyrosine kinase (Lck) recruits it to the immunological synapse to form a tri-molecular complex with CD28 [21].

PKC signaling output is exquisitely tuned to maintain cellular homeostasis, and dysregulation is associated with various diseases, including cancer and neurodegeneration [22]. To ensure proper signaling, PKC enzymes are strictly regulated by diverse mechanisms to precisely tune the amount of PKC in the cell and its signaling output. All PKC isozymes mature by a series of ordered phosphorylations that are necessary to lock the mature enzymes in an autoinhibited, but primed to signal, conformation [23,24]. In PKCθ, these phosphorylation sites correspond to Thr538 (activation loop), Ser676 (turn motif), and Ser695 (hydrophobic motif) [25–27]. Extensive studies with conventional PKC isozymes reveal that phosphorylation of the hydrophobic motif, in particular, is necessary for PKC stability, with loss of phosphate at this position triggering proteasome-mediated degradation [28,29]. Mechanisms that prevent phosphorylation, or promote the open and active conformation of PKC, trigger quality-control degradation to ensure aberrant PKC does not accumulate in the cell [30–32]. For example, cancer-associated fusion proteins in which the kinase domain of PKC is linked to other proteins, result in an open, active conformation, but this species is sensitive to dephosphorylation and degradation, resulting in paradoxical loss-of-function [33]. In cancer, conventional PKC exhibits a loss-of-function phenotype [32–35], contrasting with a gain-of-function phenotype in neurodegenerative diseases [36–39].

PKC is mutated in a variety of human cancers, making it an attractive target for drug discovery [40,41]. When it was discovered in the early 1980s that PKC is activated by tumor-promoting phorbol esters, it was characterized as an oncogene [42]. This led to the development of cancer therapies that inhibited PKC [43]. In clinical trials, all of these drugs failed to improve patient outcomes, with some worsening the disease [43]. A 2015 meta-analysis revealed a decrease in response rates and disease control in patients receiving chemotherapy in combination with PKC inhibitors compared with chemotherapy alone [44]. We now know that constitutively active PKC is susceptible to dephosphorylation and subsequent degradation, and it is the loss of PKC that promotes tumorigenesis [45,46]. In support of this, an analysis of cancer-associated mutations spanning all three PKC subclasses revealed that the majority of the mutations were loss-of-function and none were activating [34]. These data, along with patient data indicating that high levels of conventional PKC isozymes confer a survival advantage in diverse cancers [47], have reframed at least the conventional PKC isozymes as tumor suppressive. How cancer-associated mutations in PKCθ alter its function would inform on whether this novel PKC isozyme is also tumor suppressive.

Here, we analyze how four cancer-associated mutations, in three different domains, affect PKCθ activity and stability. Our results revealed that these mutations are loss-of-function by two distinct mechanisms: either by decreasing autoinhibition to promote degradation or by strengthening autoinhibition to decrease activity. Additionally, protein expression profiling reveals that PKCθ protein is reduced in diverse cancers. These results are consistent with cancer-associated mutations in PKCθ being loss-of-function, as shown previously for other PKC isozymes, and support a model in which cancer therapeutic strategies should focus on restoring PKCθ activity rather than inhibiting it.

## Results

### A multitude of mutations have been identified in PKCθ across diverse cancers

**Figure 1.**
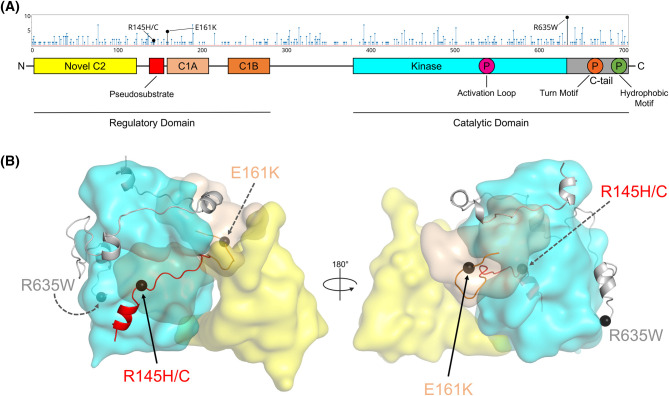
Cancer-associated mutations in PKCθ. (**A**) Mutations in PKCθ detected in human cancers and their distribution throughout the protein. Highlighted mutations are analyzed in this study. The *x*-axis depicts the position of the mutation within the full-length protein, and the *y*-axis depicts the number of patients in which the mutation was detected. Data were obtained from the cBioPortal for Cancer Genomics. (**B**) Model structure of PKCθ in its autoinhibited conformation showing the positions of the four mutations at interdomain surfaces. Mutations are predicted to disrupt autoinhibitory contacts.

To identify PKCθ mutations in human cancers, we utilized several online databases including the cBioPortal for Cancer Genomics [48–50], The Cancer Genome Atlas (TCGA), the Dependency Map (DepMap) Portal [51–56], the Catalogue of Somatic Mutations in Cancer (COSMIC) [57], and the Mitelman Database of Chromosome Aberrations and Gene Fusions in Cancer [58,59]. Our investigation revealed more than 200 unique somatic mutations in PKCθ from patient tumor samples sourced across these databases. The mutations are distributed throughout the protein, with no apparent mutational hotspots, and have been identified in a multitude of cancer types (Figure 1A). Leveraging our previously proposed model for the structure of PKCθ [17], we found that the majority of mutations participate in interdomain interactions, which we reasoned might impact autoinhibition and stability of PKC. Of these, we selected four representative cancer-associated mutations for further analysis: R145H/C in the autoinhibitory pseudosubstrate, E161K in the C1A domain, and R635W in the regulatory C-terminal tail of the catalytic domain (Figure 1B). The pseudosubstrate residue, R145, is invariant in all PKC isozymes, evolutionarily conserved, and necessary for the pseudosubstrate to bind effectively to the substrate binding cavity where it is predicted to interact with D465 and D508 in the kinase domain [32,60,61]. Mutation of this residue to His or Cys has been identified in uterine endometrioid carcinoma. The C1A domain residue, E161, whose mutation to Lys has been identified in uterine endometrioid carcinoma and cutaneous melanoma, is predicted to interact with K158 in the pseudosubstrate and T163 in the C1A domain to maintain autoinhibition. Lastly, mutation of R635 in the C-terminal tail has been identified in at least five cancer types (uterine endometrioid carcinoma, cutaneous melanoma, bladder urothelial carcinoma, rectal adenocarcinoma, and colon adenocarcinoma, with allele frequencies of 0.1–0.5). These allele frequencies indicate that for several cancers, the mutation exists in a majority of cells within the tumor. This Arg is predicted to interact with E605 and P632 in the kinase domain; most mutations are to Trp. The location of these residues at interdomain regions suggests that mutations may disrupt autoinhibitory interactions, leading to impaired PKCθ activity. As most cancer-associated mutations in PKCθ occur at predicted domain interfaces, we reason that the majority would behave in a similar manner to those analyzed.

### Cancer-associated PKCθ mutants have impaired autoinhibition

**Figure 2.**
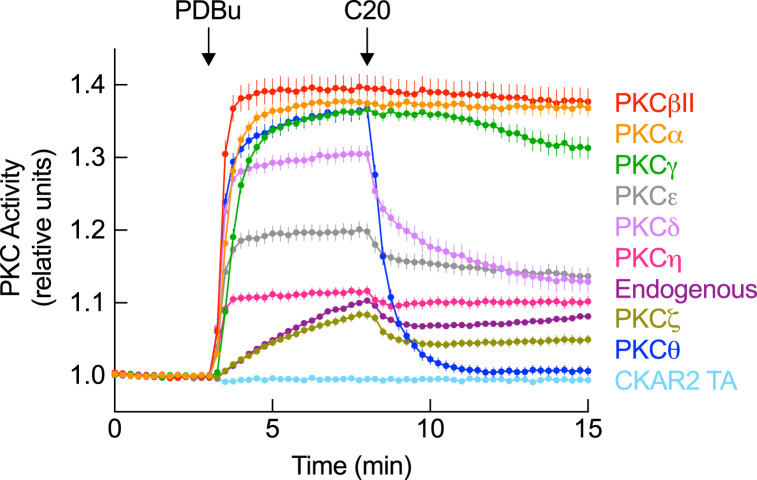
Compound 20 (C20) inhibitor is specific to PKCθ. PKC activity in COS7 cells co-expressing CKAR2 and the indicated mCherry-tagged PKC constructs. At 3 min, cells were stimulated with 200 nM PDBu followed by 1 μM of the PKCθ-specific inhibitor, C20, at 8 min. FRET/CFP ratio changes were normalized to the first 3 min and plotted. Data are representative of 37–41 cells per condition from three independent experiments (mean ± SEM). Endogenous activity was measured by transfecting cells with CKAR2 alone (dark purple trace). A nonphosphorylatable reporter (CKAR2 TA; light blue trace) was used as a control for any non-phosphorylation-dependent FRET changes.

To assess how cancer-associated mutations affected PKCθ activity, we monitored PKC activity in cells using the genetically-encoded fluorescence resonance energy transfer (FRET)-based reporter for PKC activity, C kinase activity reporter 2 (CKAR2) [62,63]; we also took advantage of the PKCθ-specific inhibitor, compound 20 (C20), to selectively inhibit PKCθ over the other PKC isozymes [64]. We first verified that C20 is indeed specific to PKCθ by systematically testing it against all conventional (α, βII, γ), novel (δ, ε, η, θ), and one atypical (ζ) PKC isozymes (Figure 2). COS7 cells expressing CKAR2 alone (representing endogenous activity, dark purple trace) or with the relevant mCherry-tagged PKC were treated with phorbol 12,13-dibutyrate (PDBu), a potent PKC agonist [42], to maximally activate PKC followed by addition of C20. CKAR2 TA (light blue trace) functioned as a non-phosphorylatable control. C20 resulted in rapid and complete inhibition of PKCθ (dark blue trace) but had no effect above that of endogenous activity on conventional PKCs (α, βII, γ), two novel PKCs (ε, η), or the atypical PKCζ. A modest effect on the paralog isozyme, PKCδ, was observed (Figure 2). Note that the slight decrease in activity in cells expressing endogenous PKC can be attributed to PKCδ, which is expressed at high levels in COS7 cells [65].

**Figure 3.**
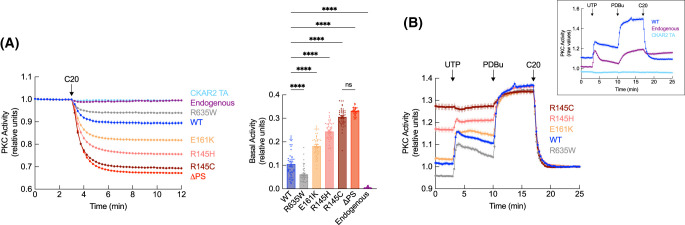
Cancer-associated PKCθ mutants have dysregulated basal activity. (**A**) Left: PKC activity in COS7 cells co-expressing CKAR2 and the indicated mCherry-tagged PKCθ constructs. At 3 min, cells were treated with 1 μM of the PKCθ-specific inhibitor, C20. FRET/CFP ratio changes were normalized to the first 3 min and plotted. Data are representative of 42–46 cells per condition from three independent experiments (mean ± SEM). Endogenous activity was measured by transfecting cells with CKAR2 alone (dark purple trace). A nonphosphorylatable reporter (CKAR2 TA; light blue trace) was used as a control for any non-phosphorylation-dependent FRET changes. Right: Basal activity was determined for each cell by subtracting the average endpoint FRET/CFP ratios from the average initial ratios of the normalized data. ns, not significant, ****P < 0.0001 by one-way ANOVA and Tukey *post hoc* test. (**B**) PKC activity in COS7 cells co-expressing CKAR2 and the indicated mCherry-tagged PKCθ constructs. At 3 min, cells were treated with 100 μM UTP, at 10 min with 200 nM PDBu, and at 17 min with 1 μM of the PKCθ-specific inhibitor, C20. FRET/CFP ratio changes were measured and plotted as raw values (insert) or normalized to the average end values once the signal stabilized. Data are representative of 39–44 cells per condition from three independent experiments (mean ± SEM). Endogenous activity was measured by transfecting cells with CKAR2 alone (dark purple trace). A nonphosphorylatable reporter (CKAR2 TA; light blue trace) was used as a control for any non-phosphorylation-dependent FRET changes.

We next assessed how each cancer-associated mutation affected the basal (unstimulated) activity of PKCθ by monitoring the magnitude of the drop in activity following PKCθ inhibition with C20. We co-overexpressed mCherry-tagged PKCθ WT, cancer-associated mutants, or a PKCθ construct lacking the pseudosubstrate (ΔPS) with CKAR2 and measured the change in FRET upon addition of C20. PKCθ WT activity was reduced upon C20 treatment, corresponding to a 68 ± 3% decrease compared with that observed for the ΔPS construct, which has been relieved of autoinhibitory constraints and represents a non-autoinhibited and constitutively active PKCθ species (Figure 3A). The pseudosubstrate mutants, R145H/C, were nearly maximally active in unstimulated cells, with no significant difference in the activities of the ΔPS mutant and the more severe R145C mutant (Figure 3A). The C1A mutant, E161K, also had an increase in basal activity, corresponding to a 45 ± 2% reduction compared with the ΔPS construct (Figure 3A). In marked contrast, the C-tail mutant, R635W, displayed a 42 ± 5% reduction in basal activity compared with PKCθ WT, reflecting enhanced autoinhibition by this mutant (Figure 3A). Thus, three mutants had reduced autoinhibition and one mutant had enhanced autoinhibition.

Next, we analyzed the agonist-evoked activity of the cancer-associated mutants. Cells were treated with [1] the natural agonist uridine-5′-triphosphate (UTP), which binds purinergic receptors to elevate second messenger, DG, and transiently activate PKC [66], to measure activation and the subsequent rate of decay (informing on how well PKCθ can re-autoinhibit), [2] PDBu to maximally activate PKC, and [3] C20 to inhibit PKCθ. mCherry-tagged PKCθ R145H/C, E161K, and R635W were co-transfected with CKAR2 in COS7 cells and sequentially treated. The FRET ratio changes were measured and plotted as raw values or normalized to the average end values once the signal stabilized. UTP stimulation resulted in a transient increase in endogenous PKC and overexpressed PKCθ WT activity that was reversed with re-autoinhibition of the enzyme following second messenger decay (Figure 3B, inset). Subsequent PDBu stimulation resulted in maximal activation of endogenous PKC and overexpressed PKCθ WT, the latter of which returned to baseline after treatment with C20 inhibitor (Figure 3B, inset). These kinetics are consistent with a properly autoinhibited PKC [67]. We then compared the activity of the mutants by normalizing the FRET ratios after inhibition. In contrast with WT, both pseudosubstrate mutants, R145H/C, were almost maximally active under basal conditions (Figure 3B). R145C did not respond to UTP stimulation and activity was only slightly increased upon PDBu treatment; R145H underwent a small increase in activity with UTP stimulation but failed to re-autoinhibit, and also underwent a modest increase in activity upon addition of PDBu. Activity of both constructs was abolished with C20 treatment (Figure 3B). The C1A mutant, E161K, showed a similar magnitude of response to both UTP and PDBu as PKCθ WT but displayed higher basal activity (as noted in Figure 3A) and a slower rate of decay (Figure 3B). The C-tail mutant, R635W, had reduced basal activity compared with WT enzyme, a reduced amplitude of activity following UTP stimulation, but the same amplitude of activity in response to PDBu (Figure 3B). The maximal activation by PDBu indicated that the mutation did not impair the intrinsic catalytic activity of PKCθ. Thus, all PKCθ mutants had altered basal activity, with mutation of R145H/C and E161K impairing autoinhibition, and R635W enhancing autoinhibition.

### PKCθ cancer-associated mutants have altered stability compared with WT

**Figure 4.**
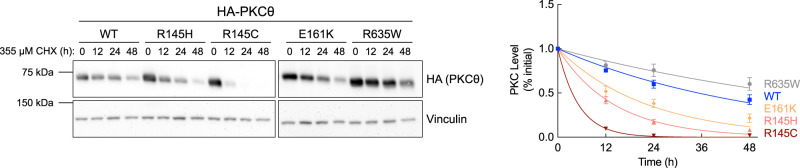
Cancer-associated PKCθ mutants show altered stability. Left: Representative Western blot of whole-cell lysate from COS7 cells transfected with the indicated HA-tagged PKCθ constructs. Cells were treated with 355 μM CHX and lysed at the indicated times. Right: Quantification of PKCθ protein levels normalized to loading control and plotted relative to the 0 h timepoint. Data were fit to a first order decay and depict mean ± SEM from at least three independent experiments.

For conventional PKC isozymes, mutants with reduced autoinhibition are less stable and turn over faster than PKC WT [32]. This is because they are in a more open conformation that is susceptible to dephosphorylation and subsequent degradation [32]. Thus, cancer-associated mutations that impair autoinhibition are paradoxically loss-of-function because the proteins are subjected to quality-control degradation [32,33]. To determine if this is the case for PKCθ, we analyzed the half-time for degradation of the enzyme in unstimulated cells. COS7 cells overexpressing HA-tagged PKCθ WT, R145H/C, E161K, or R635W were treated with cycloheximide (CHX) for up to 48 h to inhibit protein synthesis, and total PKCθ protein levels were quantified by Western blot analysis (Figure 4). PKCθ WT was relatively stable with a half-life of 37 ± 5 h. In contrast, the pseudosubstrate mutant, R145C, which abolished autoinhibition, had a half-life of 3.59 ± 0.09 h; the less severe R145H mutant had a half-life of 10 ± 1 h. The C1A mutant, E161K, which had an intermediate effect on autoinhibition, was also less stable than WT enzyme with a half-life of 16 ± 2 h. Unsurprisingly, C-tail mutant, R635W, which had enhanced autoinhibition, was more stable than PKCθ WT and had a half-life of 59 ± 8 h. These data reveal that the stability of PKCθ depends on the degree of autoinhibition. Thus, cancer-associated mutants with decreased autoinhibition, R145H/C and E161K, are susceptible to degradation, and the mutant with enhanced autoinhibition, R635W, is more stable compared with WT.

### PKCθ pseudosubstrate mutants, R145H/C, undergo rapid phorbol ester-induced down-regulation

**Figure 5.**
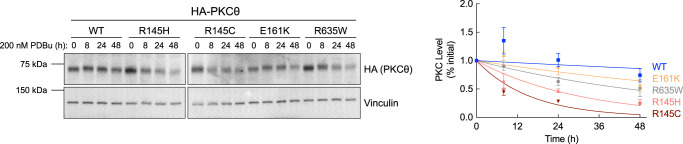
PKCθ pseudosubstrate mutants, R145H/C, undergo rapid down-regulation. Left: Representative Western blot of whole-cell lysate from COS7 cells transfected with the indicated HA-tagged PKCθ constructs. Cells were treated with 200 nM PDBu and lysed at the indicated times. Right: Quantification of PKCθ protein levels normalized to loading control and plotted relative to the 0 h timepoint. Data were fit to a first order decay and depict mean ± SEM from five independent experiments.

PKC mutants with reduced autoinhibition, or stimulated with phorbol esters, adopt an open conformation which is sensitive to dephosphorylation, ubiquitination, and degradation, a process referred to as down-regulation [24,32,34]. To assess whether the PKCθ mutants were susceptible to phorbol ester-induced down-regulation, we overexpressed HA-tagged PKCθ WT, R145H/C, E161K, and R635W in COS7 cells and treated with PDBu [31,45,68–71] for up to 48 h; whole-cell lysates were analyzed by Western blot for total PKCθ protein levels (Figure 5). Unlike conventional PKCs, which undergo rapid PDBu-mediated down-regulation, PKCθ WT was fairly resistant to PDBu with a half-time of degradation that exceeded 48 h. Notably, WT protein was up-regulated at 8 and 24 h before degrading to unstimulated levels, consistent with one previous report which showed PKCθ mRNA and protein levels increased after 20 h of PDBu stimulation [72]. In contrast, all mutants showed increased susceptibility to down-regulation. The pseudosubstrate mutants, R145C and R145H, were the most sensitive to PDBu with a half-time of degradation of 11 ± 2 h and 21 ± 2 h, respectively. The C1A mutant, E161K, which also showed an initial up-regulation followed by down-regulation at 48 h, and C-tail mutant, R635W, were also more sensitive to down-regulation compared with WT but with half-times also exceeding 48 h. The degree of sensitivity to PDBu-induced down-regulation mirrored the degree of impairment of autoinhibition for R145H/C and E161K. R635W, which has enhanced autoinhibition, also had increased sensitivity to PDBu-induced down-regulation compared with WT enzyme. This suggests that when forced into the fully-open conformation by PDBu, the mutation is recognized as aberrant and degradation is accelerated.

**Figure 6.**
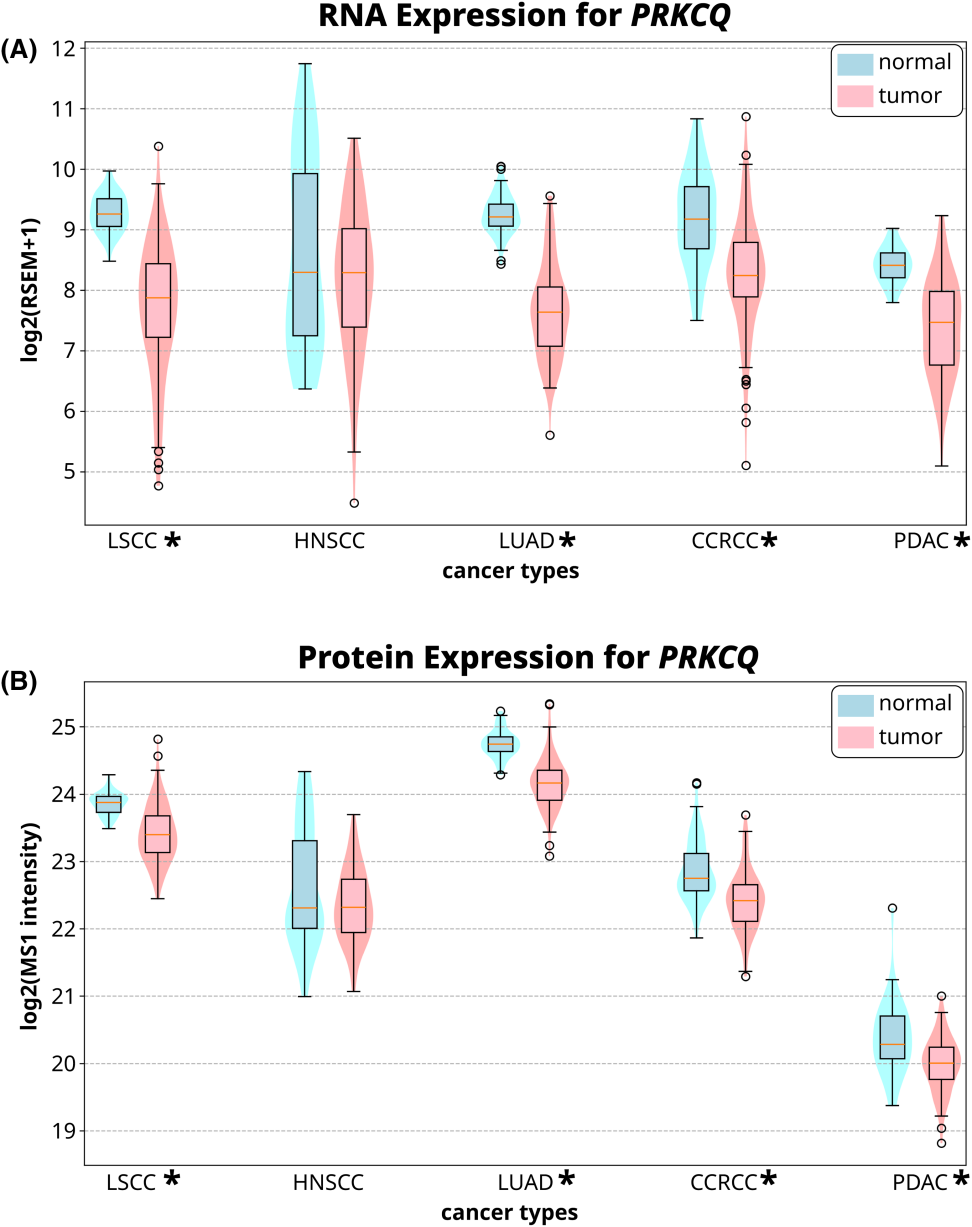
PKCθ RNA and protein expression in different cancer types. PKCθ expression levels for (**A**) RNA and (**B**) protein in five cancer types obtained from the LinkedOmicsKB database. LSCC, lung squamous cell carcinoma; HNSCC, head and neck squamous cell carcinoma; LUAD, lung adenocarcinoma; CCRCC, clear cell renal cell carcinoma; PDAC, pancreatic ductal adenocarcinoma. Boxplots depict the median (middle line) and the 25th and 75th quartiles; circles represent outliers. Color demonstrates the distribution of different samples contributing to the overall data in tumor (pink) and matched normal (blue) tissues. *P < 0.05 by Wilcoxon rank-sum test.

Our analysis indicates that cancer-associated mutations in PKCθ are likely to be loss-of-function, suggesting a tumor suppressive role. If this is the case, we reasoned that reduced PKCθ protein levels may be associated with tumor tissue compared with normal tissue. Bioinformatics analysis of multi-omics data from the LinkedOmicsKB database [73] revealed that both PKCθ mRNA (Figure 6A) and protein (Figure 6B) levels are reduced in lung squamous cell carcinoma, head and neck squamous cell carcinoma, lung adenocarcinoma, clear cell renal cell carcinoma, and pancreatic ductal adenocarcinoma compared with matched normal tissue. Thus, our mutational analysis, as well as patient data, support the hypothesis of a reduction in PKCθ function in cancer.

## Discussion

**Figure 7.**
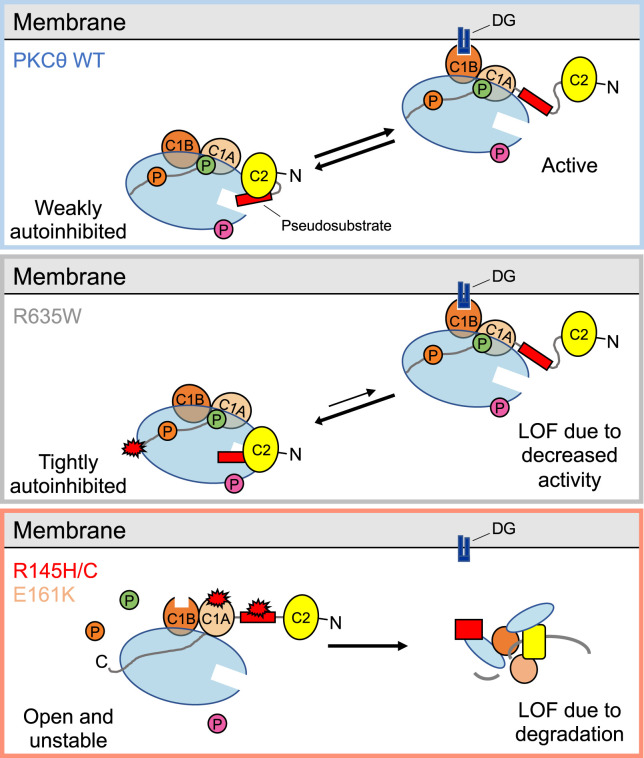
Model of PKCθ loss-of-function mutations. Top: In the absence of second messengers, PKCθ WT is maintained in a weakly autoinhibited conformation with leaky basal activity. It becomes activated by binding to second messenger, DG, at the membrane. Middle: The C-tail mutation, R635W, tightens autoinhibition, resulting in a more stable PKC that is less basally active. Bottom: Pseudosubstrate mutations, R145H/C, and C1A mutation, E161K, impair autoinhibition by disrupting interdomain interactions, resulting in a PKC with elevated basal activity that is unstable and subject to the quality-control degradation we have previously reported for Ca^2+^-regulated PKC isozymes. Thus, both types of mutations are loss-of-function, either by disrupting autoinhibition to promote degradation or strengthening autoinhibition to decrease activity.

Here, we identified two mechanisms by which cancer-associated mutations in PKCθ are loss-of-function. First, mutation of a conserved Arg in the C-tail, R635W, enhances autoinhibition to reduce the signaling output of PKCθ (Figure 7). Second, mutations in the autoinhibitory pseudosubstrate (R145H/C) and adjacent C1A domain (E161K) reduce autoinhibition such that the more active, open conformation is favored (Figure 7). This open conformation is unstable and rapidly turned over, leading to paradoxical loss-of-function. Consistent with loss of PKCθ being associated with tumors, RNA and protein expression data reveal that PKCθ levels are reduced in diverse cancers (Figure 6). Furthermore, truncating mutations as well as mutations in key catalytic residues have been detected in PKCθ, consistent with loss of PKCθ conferring a survival advantage. Taken together, our data suggest that PKCθ function is lost in cancer, and therapies should focus on restoring its activity.

In the autoinhibited conformation, the pseudosubstrate segment of PKC is tucked into the active site of the kinase domain to prevent aberrant signaling [17,74–76]. This conformation is maintained by interactions between domains [17,77–79]. Mutations that disrupt these contacts lead to defects in autoinhibition and altered basal activity [20,32–34,36–39]. Nearly all of the cancer-associated mutations detected in PKCθ occur at interdomain interfaces, including those in our analysis. R145 in the pseudosubstrate is predicted to interact with D465 and D508 in the kinase domain, E161 in the C1A domain is predicted to interact with nearby residues K158 in the pseudosubstrate and T163 in the C1A domain, and R635 in the C-tail is predicted to interact with E605 and P632 in the kinase domain. Disruption of autoinhibition is a common theme in cancer-associated mutations of other PKCs, wherein the more open conformation is subject to quality-control degradation to prevent the accumulation of aberrant PKC [32].

Of all the PKCθ mutations annotated in TCGA, the C-tail mutant, R635W, is the most frequently detected mutation in PKCθ in cancer (Figure 1A). This residue is highly conserved throughout the PKC family: conventional and atypical PKCs retain the Arg, while the other novel PKCs (δ, ε, η) harbor a Lys at this position. Furthermore, mutation of this residue has been detected in conventional, novel, and atypical PKC isozymes in cancer, underscoring its requirement for normal function. Indeed, the regulatory C-tail is frequently mutated in cancer, with mutation of the hydrophobic motif phosphorylation site, noted in PKCθ, shown to cause a reduction in the activity and a loss-of-function phenotype of PKCβII [80]. This R635W mutation in PKCθ was detected in at least five cancers, including in cutaneous melanoma where it had an allele frequency of 0.5, suggesting it is present in the melanoma cells and unlikely to be from infiltrating blood cells [48–50].

For many years, PKC was mistakenly considered an oncoprotein stemming from the fact that it is activated by tumor-promoting phorbol esters [42]. It has since been determined that prolonged activation of PKC with phorbol esters leads to its down-regulation, and rather, it is the inactivation of PKC that contributes to tumorigenesis [45,46]. Consistent with this, our analysis showed that PKCθ mutants display increased sensitivity to PDBu-mediated down-regulation compared with WT. Interestingly, PKCθ WT was considerably more resistant to PDBu-induced down-regulation compared with conventional PKC isozymes [81]. This also appears to be the case for the closely related nPKC isozyme, PKC eta (η) [81,82]. Nevertheless, the increased susceptibility of PKCθ mutants to degradation in response to PDBu is consistent with what has been observed for other PKC isozymes and support the paradigm that cancer-associated mutations in PKC are loss-of-function. Targeting the negative regulator, PH domain leucine-rich repeat protein phosphatase, represents a potential strategy to circumvent this quality-control mechanism and restore PKCθ activity [47]. Importantly, the Cancer Dependency Map (DepMap) indicates that not a single cancer cell line requires PKCθ for survival, consistent with PKCθ being tumor suppressive [51–56].

The role of cancer-associated mutations in PKCθ is less clear. Studies thus far have focused on protein expression levels, with our analysis being the first to investigate specific mutations. Furthermore, these studies have mostly been restricted to leukemia, breast cancer, and gastrointestinal stromal tumors (GISTs), although PKCθ expression in other cancers is beginning to be recognized. For example, PKCθ is expressed in pancreatic acinar cells [82] and its levels are reduced in pancreatic cancer [83]. In Notch3-dependent T-cell lymphoma, PKCθ expression was correlated with NF-κB activation and survival, with deletion of PKCθ leading to reduced incidence of disease in transgenic mice [84]. In contrast, another study reported that PKCθ-deficient mice had a higher incidence of leukemia and more rapid disease onset compared with WT mice following exposure to the Moloney-murine leukemia virus, despite having comparable leukemic cell types and similar thymus and spleen sizes [85]. These data indicate the importance of PKCθ in the immune response to leukemia. PKCθ has been shown to be highly expressed in GISTs [86], with one study reporting that PKCθ was detectable in the majority of tumors [87]. Furthermore, knockdown of PKCθ in GISTs led to a reduction in Akt activation and the up-regulation of cyclin-dependent kinase inhibitors, resulting in decreased GIST cell proliferation [88]. These data suggest that PKCθ may have an oncogenic role in these tumors. In breast cancer, PKCθ may inhibit nuclear estrogen receptor, ERα, transcription through its activation of the Akt/FOXO3a pathway, resulting in increased activity of c-Rel and expression of target genes, including c-Myc [89]. Activation of PKCθ was also associated with increased stabilization and activation of AP-1 transcription factor subunit, FRA-1, and downstream targets, leading to enhanced migration and invasion [90]. Finally, nuclear PKCθ was shown to complex with several other regulators to form a chromatin-bound active transcription complex that regulated the expression of genes involved in the epithelial-mesenchymal transition [90]. In renal cancer cells, natural compounds, tonantzitlolone and englerin A, were shown to exert a cytotoxic effect in a PKCθ-dependent manner. Both compounds activated PKCθ, resulting in heat shock factor 1 activation and insulin resistance [91,92]. Despite evidence that PKCθ is involved in cancer, its exact role remains to be elucidated. The function of PKCθ may be cancer type-specific, underscoring the need for additional analyses to determine its role in other cancers that have not yet been studied. This would be particularly informative in the clinical setting where PKCθ-specific inhibitors are in the early stages of development, primarily for autoimmune and inflammatory diseases [93]. Given our findings presented here, it is worth considering whether these compounds might pose a risk for cancer.

It is worth noting that without more advanced studies, it is difficult to distinguish whether the aberrant PKCθ is expressed in tumor cells or the tumor immune microenvironment. It is well documented that PKCθ is selectively expressed in hematopoietic cells and, furthermore, plays a role in the immune response to cancer [15,94,95]. In particular, the CD28 co-stimulatory pathway has been shown to play a pivotal role in blocking immune checkpoints, thus enhancing anti-tumor responses [96]. Given the central role of PKCθ in mediating CD28 signaling, it stands to reason that PKCθ also plays an essential role in this process. Consistent with this, one study reported that *PRKCQ* expression was positively correlated with tumor infiltrating lymphocytes, in the majority of cancer types analyzed, and was associated with improved patient survival [15]. Moreover, PKCθ-deficient T cells failed to become activated due to impaired transcription factor activation and IL-2 secretion [14,97–99]. Our analysis shows that cancer-associated mutations in PKCθ lead to a loss-of-function phenotype; however, whether these impact disease progression directly or indirectly due to impaired immune responses remains to be determined.

Mutations in PKCθ have been detected in lymphomas and leukemias, including 2 fusion proteins (*FAM107B-PRKCQ* and *PTGIS-PRKCQ*, respectively), with two additional fusion proteins being detected in breast and ovarian cancers [58,59]. We have previously reported that for PKCθ fusion proteins, the 3′ end of PKC is retained while the 5′ regulatory region is lost [33]. This is a clear example of PKCθ not being able to autoinhibit due to the absence of its N-terminal regulatory domains and would be expected to behave in a manner similar to the PKCθ R145H/C pseudosubstrate mutants. Our prior analysis of 3′ PKC fusion proteins in a conventional PKC revealed these to be loss-of-function due to decreased stability that prevents the accumulation of the protein in cells [33].

In summary, our analysis of cancer-associated mutations in PKCθ reveals two distinct mechanisms by which signaling output is impaired — either by enhancing autoinhibition or by disrupting autoinhibition to promote degradation and loss of protein (Figure 7). These data suggest that the quality-control mechanisms that prevent aberrant conventional PKC from accumulating in cells also pertain to PKCθ. In addition to the domain interface mutations we explored, several residues in the kinase domain involved in catalysis are mutated (e.g. T538 in the activation loop, G524 in the DFG motif, and R503 in the HRD motif). Note that mutations in the C1 domains are underrepresented in cancer [37] as these can stabilize the mutant protein; indeed, the C1 domains are mutational hotspots in the neurodegenerative disease, spinocerebellar ataxia type 14, which is caused by impaired autoinhibition of PKCγ [37]. Given the loss of PKCθ protein in diverse cancers, these results suggest that restoring PKCθ function may be the relevant strategy for cancer therapies.

## Materials and methods

### Identification of PKCθ cancer-associated mutations

Mutations in PKCθ were identified from the following online databases: the cBioPortal for Cancer Genomics [48–50], TCGA, the Dependency Map (DepMap) Portal [51–56], the COSMIC [57], and the Mitelman Database of Chromosome Aberrations and Gene Fusions in Cancer (https://mitelmandatabase.isb-cgc.org/) [58,59].

### Plasmids and constructs

The CKAR2 and CKAR2 TA were previously described [63]. Human PKCθ was N-terminally tagged with mCherry and HA in a pcDNA3 vector using Gateway cloning (Life Technologies) as previously described in [34]. All mutants were generated using QuikChange site-directed mutagenesis (Agilent) following the manufacturer's instructions.

### Antibodies and reagents

Vinculin antibody (cat. no. 4650S) was from Cell Signaling Technologies and HA antibody (cat. no. H9658) was from Millipore and were used at 1:1000 dilution. HRP-conjugated anti-rabbit (cat. no. 401315) and anti-mouse (cat. no. 401215) secondary antibodies were from Millipore and used at 1:10 000 dilution. All antibodies were diluted in 1% BSA (Millipore, cat. no. 12659) dissolved in PBS-T (1.5 mM Sodium Phosphate Monobasic, 8 mM Sodium Phosphate Dibasic, 150 mM NaCl, 0.05% Tween-20) with 0.25 mM thimerosal (Thermo Scientific, cat. no. J61799.14). UTP (catalog no. 6701) and PDBu (cat. no. 524390) were purchased from Calbiochem. Compound 20 (C20) (cat. no. S6577) was purchased from Selleckchem. Cycloheximide (cat. no. 239763) was purchased from Millipore. Bradford reagent (cat. no. 500-0006), protein standards (cat. no. 161-0394), bis/acrylamide solution (cat. no. 161-0156), and polyvinylidene difluoride (PVDF) membrane (cat. no. 162-0177) were purchased from Bio-Rad. Luminol (cat. no. A-8511) and p-coumaric acid (cat. no. C-9008) used to make chemiluminescence solution were purchased from Sigma–Aldrich.

### Cell culture and transfection

COS7 cells were maintained in Dulbecco's modified Eagle's medium (Corning, cat. no. 10-013-CV) containing 10% fetal bovine serum (Atlanta Biologicals, cat. no. S11150) and 1% penicillin/streptomycin (Gibco, cat. no. 15-140-122) at 37°C in 5% CO_2_. Cells were periodically tested for Mycoplasma contamination by a PCR-based method [100]. Transient transfections were carried out using a Lipofectamine 3000 kit (Thermo Fisher Scientific) per the manufacturer's instructions, and constructs were allowed to express for 24 h for imaging experiments and CHX assays or for 48 h for PDBu assays.

### FRET imaging and analysis

COS7 cells were seeded into 35 mm dishes (Corning, cat. no. 430165) containing glass cover slips (Fisherbrand, cat. no. 12545102) attached with SYLGARD 184 Silicone Elastomer Kit (Dow, cat. no. 04019862). Cells were transfected 24 h after seeding. For CKAR2 activity assays, cells were co-transfected with 1 μg mCherry-PKC constructs and 1 μg CKAR2 or CKAR2 TA DNA [63]. Cells were imaged 24 h post-transfection. Cells were rinsed once and imaged in 2 ml Hank's Balanced Salt Solution (Corning, cat. no. 21-022-CV) with 1 mM CaCl_2_ added fresh prior to imaging. Images were acquired on a Zeiss Axiovert 200 M microscope (Carl Zeiss Micro-Imaging Inc.) using an Andor iXonUltra 888 digital camera (Oxford Instruments) controlled by MetaFluor software (Molecular Devices) version 7.10.1.161. Filter sets and parameters for imaging were described previously [101]. Background signal was subtracted for each wavelength from an area containing no cells. Individual cells were selected for quantification by tracing the entirety of the cell. Images were acquired every 15 s, and baseline images were acquired for 3 min prior to drug addition. 100 μM UTP, 200 nM PDBu, and/or 1 μM C20 diluted in imaging solution were added dropwise to the dish in-between acquisitions. FRET ratios for each cell were normalized to the average of the first or last 3 min unless otherwise indicated in the figure legends. Basal activity was calculated for each cell by subtracting the average of the last 3 min from the average of the first 3 min. Data represent mean ± SEM for cells from at least three independent experiments.

### Cell lysis and immunoblotting

Cells were washed with Dulbecco's phosphate-buffered saline (Corning, cat. no. 21-031-CV) and lysed in Phosphate Lysis Buffer pH 7.5 containing 50 mM Na_2_HPO_3_, 1 mM Na_4_P_2_O_7_, 20 mM NaF, 2 mM EDTA, 2 mM EGTA, and 1% Triton X-100. Lysis buffer was supplemented with 50 μg/ml leupeptin, 1 mM PMSF, 1 mM Na_3_VO_4_, 2 mM benzamidine, 1 mM DTT, and 1 μM microcystin prior to lysis. Whole-cell lysates were collected by scraping and briefly sonicated prior to protein quantification by Bradford Assay. Samples were boiled in sample buffer containing 250 mM Tris HCl, 8% (w/v) SDS, 40% (v/v) glycerol, 80 μg/ml bromophenol blue, and 2.86 M β-mercaptoethanol for 5 min at 95°C. 20–30 μg protein per sample was analyzed by SDS–PAGE using 7–8% acrylamide gels. Gels were transferred to PVDF membranes by wet transfer method at 4°C for 2 h at 80 V in transfer buffer (200 mM Glycine, 25 mM Tris–HCl, 20% Methanol). Membranes were blocked in 5% milk dissolved in PBS-T for 30 min at room temperature. Membranes were then washed with PBS-T for 5 min three times and incubated with primary antibody overnight at 4°C with rocking. Membranes were washed for 5 min three times in PBS-T, incubated in the appropriate secondary antibodies for 1 h at room temperature, washed for 5 min three times in PBS-T, and developed with chemiluminescent substrate (100 mM Tris–HCl (pH 8.5), 1.25 mM luminol, 198 μM coumaric acid, and 1% H_2_O_2_) on a FluorChem Q imaging system (ProteinSimple).

### CHX assay

COS7 cells were seeded into six-well plates at 2 × 10^5^ cells per well. After 24 h, cells were transfected with 1 μg of the indicated HA-tagged PKCθ constructs and allowed to incubate for 24 h before CHX treatment. Cells were treated with 355 μM CHX or dimethyl sulfoxide (DMSO) control, lysed at the indicated times, and analyzed by Western blot.

### Phorbol ester down-regulation assay

COS7 cells were seeded into six-well plates at 2 × 10^5^ cells per well. After 24 h, cells were transfected with 500 ng of the indicated HA-tagged PKCθ constructs. Twenty-four hours of post-transfection, the media was replaced with fresh Dulbecco's modified Eagle's medium containing 10% fetal bovine serum and 1% penicillin/streptomycin, and cells were allowed to incubate for an additional 24 h before PDBu treatment. Cells were treated with 200 nM PDBu or DMSO control, lysed at the indicated times, and analyzed by Western blot.

### Bioinformatics analysis

#### Data collection, preprocessing, and mapping of mutational data on PKCθ’s full protein sequence

The mutational data for PKCθ were derived from TCGA PanCancer Atlas Study [PMID: 29288495], which was retrieved using the ‘Quick Select’ feature in the cBioPortal (v6.0.2) website [PMID:22588877]. A ‘Query By Gene’ using the ‘PRKCQ’ gene name was used to retrieve all PKCθ associated mutations from the 32 TCGA PanCancer Atlas Studies. Each mutation was mapped to the full-length sequence and frequency of mutation at each position was visualized using a stem plot created using the Python matplotlib package (version 3.8.1).

#### Data collection and preprocessing for the RNA and protein expression of PKCθ

Normalized RNA and protein expression data for PKCθ were obtained from the LinkedOmicsKB Release 1 (05/08/2023) [PMID: 37619559] and preprocessed using native Python libraries (version 3.9.17). RNA expression levels are indicated in log_2_(RSEM + 1) units and protein expression levels are indicated as log_2_(MS1 intensity). To ensure proper controls, only cancer types with data for both tumor and normal samples were included in the analysis. This filtering step resulted in five cancer types containing both RNA and protein expression of PKCθ in tumor-normal sample pairs.

#### RNA and protein expression visualization and statistical analysis of PKCθ

The RNA and protein expression of PKCθ in the five cancer types are represented as overlayed boxplots and violin plots. Both plots were generated using the Python package matplotlib (version 3.8.1). The statistical significance of RNA and protein expression between tumor and normal samples was estimated using a Wilcoxon rank sum test using the SciPy Python package (version 1.12.0).

### Quantification and statistical analysis

For imaging experiments, intensity values and FRET ratios were acquired using MetaFluor software and normalized as described above. Western blots were quantified by densitometry using ImageJ version 2.1.0/1.53c. Statistical tests described in figure legends were performed using Prism (GraphPad Software) version 9.5.0. For the CHX assay, outliers were identified using the Grubbs’ test (*α* = 0.01) in Prism and removed. Structures were modeled using PyMOL version 2.3.0 (Schrödinger, LLC).

## Data Availability

The findings of this study are supported by the data within the article.

## Open Access

Open access for this article was enabled by the participation of University of California in an all-inclusive *Read & Publish* agreement with Portland Press and the Biochemical Society under a transformative agreement with UC.

## Abbreviations

C20: compound 20
CHX: cycloheximide
CKAR2: C kinase activity reporter 2
COSMIC: Catalogue of Somatic Mutations in Cancer
DG: diacylglycerol
FRET: fluorescence resonance energy transfer
GIST: gastrointestinal stromal tumor
nPKC: novel protein kinase C
PDBu: phorbol 12,13-dibutyrate
PKC: protein kinase C
PVDF: polyvinylidene difluoride
TCGA: The Cancer Genome Atlas
UTP: uridine-5′-triphosphate
